# Primary and secondary prevention of cardiovascular disease in patients with hyperlipoproteinemia (a)

**DOI:** 10.1007/s11789-017-0090-3

**Published:** 2017-02-23

**Authors:** P. Grützmacher, B. Öhm, S. Szymczak, C. Dorbath, M. Brzoska, C. Kleinert

**Affiliations:** 2nd Medical Clinic – Nephrology, Hypertension and Vascular Diseases, AGAPLESION Markus-Hospital, Frankfurt/Main, Germany

**Keywords:** Lipoprotein (a), Lp (a) apheresis, Cardiovascular prevention, Target LDL cholesterol

## Abstract

General lipoprotein (Lp) (a) screening can help to identify patients at high risk for cardiovascular disease. Non-invasive methods allow early detection of clinically asymptomatic incipient atherosclerotic disease. Medical treatment options are still unsatisfactory. Lp(a) apheresis is an established treatment in Germany for secondary prevention of progressive cardiovascular disease. Statin-based lowering of LDL cholesterol and thrombocyte aggregation inhibitors still represent the basis of medical treatment. Target levels for LDL-cholesterol should be modified in patients with hyperlipoproteinemia (a).

## Introduction

The role of Lp (a) as an independent risk factor is meanwhile generally accepted [1–3]. The aim of secondary prevention of cardiovascular and other vascular diseases in patients with hyperlipoproteinemia (a) is to prevent further lethal and non-lethal complications, if an atherosclerotic disease is already clinically manifest. Mostly the coronary arteries, the arteries of the lower extremities and the cerebrovascular system of patients in the second half of life are involved. Primary prevention usually focuses on younger patients without clinically symptomatic atherosclerotic disease. Statins have shown to be effective in primary prevention even in patients with intermediate risk [4, 5].

The use of non-invasive diagnostic procedures as e. g. B‑mode sonography of blood vessels or cardiac computed tomography contributes to early risk stratification. With these techniques a continuous progression of atherosclerotic plaques sometimes can be observed over decades in clinically asymptomatic patients. Therefore primary and secondary prevention are no longer strictly discriminated.

## Indication for screening of Lp (a)

As screening for lipoprotein (Lp) (a) of the general population is currently not yet recommended, many patients miss early preventive strategies. For secondary prevention, Lp (a) should be measured in premature cardiovascular disease and progressive atherosclerotic disease despite correction of all other risk factors, especially despite optimal lipid-lowering treatment. For primary prevention, Lp (a) screening is recommended in patients with a positive family history of premature cardiovascular diseases, elevated Lp (a) in other family members, familial hypercholesterolemia, and in high-risk patients with a 10-year risk of fatal cardiovascular disease of 5–10% according to the ESC score [6]. It should be discussed to extend Lp (a) screening to every individual with a vascular event, which can not sufficiently be explained by typical risk factors, independent of the patient’s age. Furthermore, a high coincidence with genetically induced hemostatic defects has to be considered [7].

End-stage renal disease and the nephrotic syndrome are most frequent causes of secondary hypolipoproteinemia (a) [8, 9].

In many patients, an unexpected cardiovascular event induces the first measurement of Lp (a) and a profound evaluation of conventional, generally accepted risk factors; the German lipid league proposes a general screening of the whole population by at least one single measurement in life. As the laboratory methods still have a high variance, 2–3 controls may be indicated, if exact risk estimation is necessary [9, 10].

## Therapeutic options in hyperlipoproteinemia (a)

Lifestyle changes and statins have no relevant effects on serum Lp (a) concentrations. Several drugs are able to reduce elevated Lp (a) levels by 5–30%. However, up to now there is no evidence of any reduction of clinical vascular endpoints for all substances. Neither has any of these drugs been approved by the German authorities for the treatment of hyperlipoproteinemia (a) (Table 1).

**Table 1:** Drugs with significant effects on serum Lp(a) concentration

Substance	Mode of action	Reduction of Lp(a) (%)	Special notes
Nicotinic acid	Classical drug	20–30	Moderate side effects
Evolocumab Alirocumab	PCSK9 antibodies	15–30	Very low side effects
Lomitapide	MTP inhibitor	15–32	Risk of steatosis
Mipomersen	Apo B100 antisense oligonucleotide	20–35	Risk of steatosis
ISIS-APO (a) 144367	Apo (a) antisense oilgonucelotide	30–80	Clinical trials still running

No drug has yet been approved for specific treatment of hyperlipoproteinemia (a)

No effect on clinical endpoints has yet been demonstrated in neither drug

**Table 2:** Primary and secondary prevention of cardiovascular disease in patients with hyperlipoproteinemia (a). Possible therapeutic strategies

	Age years	Lifestyle changes	Target LDL-chol	Statins	Platelet inhibition (aspirine)	Additional options
Primary prevention	<35	+++	<115 mg%	–	–	Correct triglycerides
	>35	++	<100 mg%	+	50 mg/d?	″
	<60	++	<100 mg%	+	50 mg/d	″
Secondary Prevention	<60	++	<50 mg%	++	100 mg/d	″
	>60	+	<70 mg%	++	100 mg/d	″
Progression	–	+	<30 mg%	++	100 mg/d	″
(Lp(a) apheresis obligatory)						Consider dual platelet inhibition, anticoagulation as last option?

Nicotinic acid at a daily dose of 2–3 g/die can reduce Lp (a) levels by up to 30%. Similar results have been shown for microsomal triglyceride transfer protein inhibitor lomitapide and the apo-B-100 antisense oligonucleotide mipomersen. However, both drugs bear a considerable risk of the development of fatty liver disease, being the main reason of failing German drug approval for the treatment of elevated LDL-cholesterol and lipoprotein (a) levels [11–13].

Two PCSK9-antibodies have been introduced for the treatment of severe hypercholesterolemia, refractory to conventional drug combinations. In contrast to their impressive potential on LDL-cholesterol, the influence on Lp (a) is markedly lower; a lowering of Lp (a) levels by up to 30% has been reported, the reduction rate is below 20% in patients with high levels of Lp (a) [14, 15].

A most promising approach is the antisense oligonucleotide against apolipoprotein (a), where reduction rates up to 80% seem possible; nevertheless, the necessary clinical study protocols for drug approval have not yet been completed [16]. Therefore, in daily practice no option for a direct medical correction of hyperlipoproteinemia (a) is available.

In Germany Lp (a) apheresis is an established treatment for patients with elevated Lp (a) levels providing reduction rates of 60–70% compared to baseline and pre-apheresis levels. Lp (a) apheresis has been approved for secondary prevention in patients with clinically manifest cardiovascular diseases, which is progressive despite the correction of all other risk factors, and in patients with already extended cardiovascular diseases, in whom a progression is assumed to have deleterious consequences [17].

An impressive reduction of cardiovascular complications has been observed in five observation studies in different German patient cohorts [18–23].

The annual quality report of the Kassenärztliche Bundesvereinigung of 2015 included 953 patients with isolated hyperlipoproteinemia (a) treated with regular Lp (a) apheresis [24].

## Treatment of hyperlipoproteinemia (a) by individual risk stratification

Without any effective medical treatment option for lowering Lp(a)-levels, primary prevention has to focus on on the reduction of the total individual risk for cardiovascular disease and thus on the correction of classical concomitant risk factors which are not discussed here (Table 2).

In young and healthy patients without risk factors, even strongly elevated Lp (a) levels to more than 3.5fold above normal induce only a small increment of the absolute cardiovascular risk – in spite of doubling the relative risk. However, if other factors such ass smoking, hypertension, male sex, age >60 years or classical Framingham risks of >20%/10 years are present, a dramatic increment of the absolute risk can been observed [25, 26].

But the risk of elevated Lp (a) level alone is already comparable to the risk of smoking or arterial hypertension in low risk situations, those being classical targets of preventive efforts in daily practice.

Further discrimination of cardiovascular risk is partially possible by the measurement of the genetic variants rs10455872 and 3798220, which determine the serum concentration of Lp (a) as well as the size of Lp (a) particles by the numbers of kringle IV-type 2 copies [27].

Except for apheresis, specific recommendations for the management of patients with hyperlipoproteinemia (a) have not yet been established. This is explained by the lack of therapeutics options and of clinical evidence of any differentiated medical strategy.

It has been shown that the cardiovascular risk of elevated of LDL cholesterol is considerably increased in the presence of an additionally elevated Lp(a) level (a) [28, 29].

It is the current concept to establish optimal LDL-cholesterol target levels in patients with hyperlipoproteinemia (a) by means of dietary restrictions and the use of statins, although this strategy has not yet been confirmed by clinical endpoint studies [30].

In patients with moderate risk (score risk 1 ≤ 5%), the current ESC/EAS-guideline of 2016 recommend a target LDL-cholesterol of <115 mg% (<3.0 mmol/l) if at least one classical major risk factor is present (6). Although Lp(a) is not yet accepted as a major risk factor, this target level should be implemented for patients with elevated Lp(a)-levels.

In patient with a 10-year risk of 5 ≤ 10%, ESC/EAS-guidelines recommend a LDL target level of <100 mg%, if at least one further major risk factor is present. It should be remembered that the risk difference of these 2 groups is mainly caused by gender and age.

The use of platelet inhibition is not generally recommended for primary prevention even in elderly persons [40]. As Lp (a) exerts considerable prothrombotic effects [31, 32], a primary protection can be discussed, e. g. using low dose aspirin in adult patients >35 years of age. At least in patients >60 years, a positive risk/benefit ratio may be expected, if already minor evidence of vessel alterations is present.

In patients with clinically symptomatic atherosclerotic disease, secondary prevention regularly includes the use of platelet inhibitors, usually aspirin at a dose of 100 mg/die and the use of statins in order to reduce LDL-cholesterol below a target level of 70 mg% [33–36].

In Lp(a) patients with premature cardiovascular disease below 60 years of age, a therapeutic target of <50 mg% may be regarded as a more safe strategy, and in patients with advanced or progressive cardiovascular disease despite optimal guideline-based therapy, aggressive lowering of LDL-cholesterol to <30 mg% as well as combined platelet inhibition should be considered, as these regimens hardly bear any clinically relevant risk [37, 38].

Apart from that, the correction of elevated serum triglycerides should further contribute to a reduction of the total cardiovascular risk [39].

In exceptional cases with advanced and recurrent vascular occlusions, triple therapy including anticoagulatory substances as vitamin K antagonists, thrombin and factor Xa inhibitors may be advantageous [38, 40].

## Conclusion

In patients with hyperlipidaemia (a) very early identification and comprehensive risk management are mandatory for successful cardiovascular prevention as long as a direct and efficient medical correction is not available and Lp(a) apheresis is not yet required.
